# Pathology-Dependent Histological Changes of the Left Stellate Ganglia: A Cadaveric Study

**DOI:** 10.4137/cpath.s979

**Published:** 2008-10-30

**Authors:** Salvatore Docimo, Carmen Piccolo, Daniel Van Arsdale, David E. Elkowitz

**Affiliations:** 1 Academic Medicine Fellow, Department of Pathology, New York College of Osteopathic Medicine, Old Westbury, NY; 2 Resident, Department of Surgery, Philadelphia College of Osteopathic Medicine, Philadelphia, PA; 3 Department of Osteopathic Manipulative Medicine, New York College of Osteopathic Medicine, Old Westbury, NY; 4 Department of Pathology, New York College of Osteopathic Medicine, Old Westbury, NY

**Keywords:** stellate ganglion, sympathetic hyperinnervation, nerve sprouting, cardiopulmonary

## Abstract

Sympathetic hyperinnervation due to nerve sprouting generated by the left stellate ganglion has been noted following cardiopulmonary disease processes. Sympathetic hyperinnervation seems to be limited to cardiopulmonary diseases in the experimental and clinical settings. However, histological changes of the left stellate ganglion following cardiopulmonary diseases in humans have vet to be observed. This study intends to investigate the histological changes of cadaveric sympathetic nervous tissue of left stellate ganglia (n = 32) and their relationship to noted pathology. Our study found fibrotic changes of the left stellate ganglion are not significantly dependent upon pathological processes, however, changes in the number of nerve cell bodies seems to be pathology dependent. A relationship between respiratory (mean = 33.3; P = 0.023) and cardiovascular pathologies (mean = 29.6; P = 0.199) and an increase in nerve cell bodies of the left stellate ganglion was noted when compared to other pathologies (mean = 25.7). The link between cardiopulmonary disease and sympathetic hyperinnervation may be the increase in the number of nerve cell bodies of the left stellate ganglion. Our results are clinically significant considering sympathetic hyperinnervation is associated with arrythmogenesis and an increase in morbidity and mortality in patients with pulmonary disease. Such findings may warrant investigation into the use of ganglion blockade in cardiopulmonary diseases.

## Introduction

Recent evidence shows that the autonomic nervous system (ANS) may play a role in the morbidity and mortality of certain pathological states. For instance, the level of Sympathetic activation has been associated with arrhythmic complications, such as ventricular arrhythmias (Jayachandran et al. 2000; Gould et al. 2003). Although designed to meet the body’s demands in times of stress, the ANS system may contribute to disease processes when inappropriately maintained.

Sympathetic stimulation has been linked to a variety of cardiovascular pathologies. The level of Sympathetic activation has been shown to correlate with arrhythmic complications (Jayachandran et al. 2000; Gould et al. 2003). Post-myocardial infarction (Post-MI) sympathetic hyperinnervation has been associated with the generation of ventricular arrhythmias leading to sudden cardiac death (SCD) (Swissa et al. 2004) and an increased incidence of atrial fibrillation (AF) (Miyauchi et al. 2003; Swissa et al. 2005). Ischemic injury due to a MI has also been shown to cause nerve degeneration followed by sympathetic regeneration (nerve sprouting) and hyperinnervation to the atria leading to AF (Miyauchi et al. 2003).

Ventricular arrhythmias have also been associated with post-MI sympathetic hyperinnervation in pathological specimens from native hearts of cardiac transplant recipients (Cao et al. 2000). The density of S100-immunoreactive nerve fibers was significantly higher in the periphery of injured ventricular myocardium in individuals with a history of arrhythmias than in those without arrhythmias (Cao et al. 2000). In a canine post-MI model, the infusion of nerve growth factor (NGF) into the left stellate ganglion (LSG) accelerated the development of nerve sprouting, created sympathetic nerve densities similar to the nerve densities of the cardiac transplant specimens, and resulted in increased incidence of ventricular tachycardia and SCD (Cao et al. 2000). These findings are suggestive of an association between post-injury sympathetic nerve density and an increased susceptibility to life-threatening arrhythmias (Verrier and Kwaku, 2004).

In addition, hyperinnervation of the sympathetic nervous system has been observed in respiratory pathology. Excess sympathetic activity has been described as a cause of increased pulmonary vascular resistance and implicated in the disease processes of cor pulmonale and pulmonary hypertension (Heindl, 2001). More commonly, ailments such as chronic obstructive pulmonary disease (COPD) have also been linked to an increased constant sympathetic signal (Volterrani et al. 1994; Scalvani et al. 1999; Bartels et al. 2000).

Clinical and experimental evidence linking the LSG to cardiopulmonary diseases prompted us to evaluate its histological changes under certain disease states. Evidence such as the association between infusion of NGF into the LSG and ventricular tachycardia and fibrillation (Verrier and Kawaku, 2004) a nerve sprouting signal from the LSG triggering a generalized increase in cardiac nerve density throughout the heart (Zhou et al. 2004), and the fact the LSG houses the cells that supply the cardiopulmonary system (Pardini et al. 1990).

## Materials and Methods

### Sample collection methods

Left stellate ganglia were removed from randomly selected human cadavers of a New York state donor population at the New York College of Osteopathic Medicine. Age range of the cadavers was 28 to 98 with a mean of 78. Cadavers were placed in the supine position with their necks positioned in extension. Initial incisions were made from the angle of the mandible to the sternal notch and to the distal portion of the clavicle. Muscles such as the platysma muscle and sternocleidomastoid muscle were removed or reflected. Once the vasculature was exposed, the left stellate ganglia could be found on the sympathetic chain deep to the point at which the left internal thoracic artery branches from the left subclavian artery. The left stellate ganglion is formed by the fusion of the inferior cervical ganglion with the first thoracic ganglion.^18^ Left stellate ganglia (n = 32) were detached from the sympathethic chain using a scalpel and forceps and then stored in three times the specimen volume in 10% buffered formalin.

### Histological methods

Ganglia were serially sliced along the vertical plane into 2 mm thick sections, trimmed, and placed into cutting blocks. The specimens were placed in 10% buffered formalin for 24 hours followed by treatment with 70%–100% ethanol and xylene. Specimens were embedded in paraffin and sectioned at a thickness of 6 μm via a microtome. Four slides were stained for each specimen, two with hematoxylin and eosin, and two with Masson’s trichrome stain. Slides were observed with an Olympus BX41 multihead microscope and digitally photographed.

Based on cadaver’s cause of death, ganglia were grouped into one of three categories: cardiovascular disease, respiratory disease, and all other pathological processes excluding the cardiopulmonary system. Each slide was examined to determine the percentage of fibrosis and an estimated amount of nerve cell bodies per ganglia.

The percentage of fibrosis was quantified using a grading scale created against a control ganglion that exhibited minimal fibrosis. The control ganglion was determined by a pathologist to be the ganglion with the least amount of fibrotic changes in comparison to all other specimens. Changes in nerve cell bodies were determined by counting the number of nerve cell bodies present in three separate high-powered fields at a 40x magnification. The number of nerve cell bodies calculated in each of the three fields was then averaged.

### Statistical analysis

Student’s t tests were used to compare the means between two groups. The number of ganglion cells per three high powered fields was average and this mean was used in the analysis of this variable. The t tests were used to compare the means of the following grouping categories: fibrosis (%) of cardiovascular samples vs. fibrosis (%) of other pathologies; fibrosis (%) of respiratory samples vs. fibrosis (%) of other pathologies: nerve cell bodies average of cardiovascular samples vs. nerve cell bodies average of other pathologies; nerve cell bodies of respiratory samples vs. nerve cell bodies of other pathologies. A p value ≤ 0.05 was considered significant.

## Results

### Fibrotic chances of the left stellate ganglia

**Figure 1.**
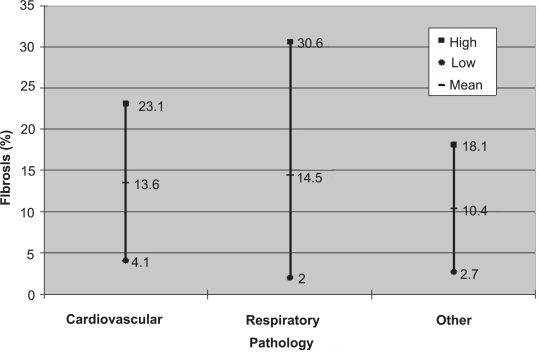
**Observed fibrosis**. Mean and standard deviation of fibrosis (%) observed within left stellate ganglia based upon pathologies.

The percentage of fibrosis among cadavers with cardiovascular disease (n = 12) had a mean fibrosis of 13.6%, cadavers with respiratory disease pathology (n = 10) had a mean fibrosis of 14.5%, and cadavers with all other pathology (n = 10) had a mean fibrosis of 10.4% (Fig. 1). Table 1 describes the pathologies that fall under each category. The difference in mean fibrosis between cadavers with cardiovascular disease and cadavers with all other pathology was not statistically significant (p = 0.396). The difference in mean fibrosis between the cadavers with respiratory disease pathology and cadavers with all other pathology was not statistically significant (p = 0.272).

### Nerve cell body changes of the left stellate ganglia

**Figure 2.**
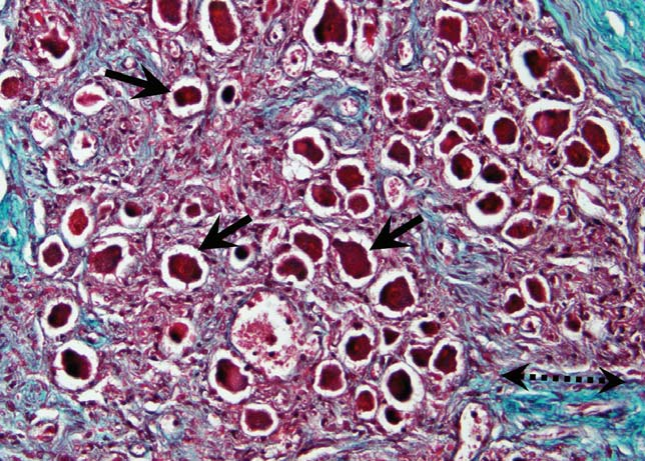
**Stellate ganglion section: Cardiovascular disease**. Histological section of the left stellate ganglion stained with Masson’s trichrome stain. Sample was removed form a cadaver with a history of coronary heart disease and atrial fibrillation. Note increased number of nerve cell bodies (arrows). Light blue area indicates fibrosis (dashed-line double-headed arrow). Note the areas of fibrosis do not infiltrate the nerve cell bodies.

The mean nerve cell bodies per field at a power of 40x per left stellate ganglion for cadavers with cardiovascular disease (n = 12) was 29.6, the mean nerve cell bodies for cadavers with respiratory disease (n = 10) was 33.3, and the mean nerve cell bodies for cadavers with all other pathology (n =10) was 25.7 (Fig. 2). Table 1 describes the characteristics and pathology distribution of each cadaver. The difference in mean nerve cell bodies per high powered field between cadavers with cardiovascular disease and cadavers with all other pathology was not statistically significant (p = 0.199). The difference in mean nerve cell bodies for cadavers with respiratory disease pathology and cadavers with all other pathology was found to be statistically significant (p = 0.023).

## Discussion

Strong evidence linking the LSG to cardiopulmonary diseases lead us to focus our study on the LSG only. Stimulation of the LSG has lead to prolongation of the Q-T interval which can lead to arrhythmia, whereas, the right stellate ganglion affected the Q-T interval insignificantly (Wong, 1997). Stimulation of the left stellate ganglion has continually demonstrated an increase in the generation of arrhythmias (Yanowitz et al. 1966; Verrier et al. 1974; Schwartz et al. 1985; Janse et al. 1985; Schwartz et al. 1990; Schwartz et al. 1998). The LSG’s involvement in pulmonary pathology has also been supported. Pulmonary edema (Theodore and Robin, 1976; Ingram and Braunwald, 1980) and pulmonary vasoconstriction, both of which can worsen pathological processes of the respiratory system, have each been associated with increased sympathetic signals (Sarnoff and Sarnoff, 1953; Szidon et al. 1971; Theodore and Robin, 1976; Ingram and Braunwald, 1980). Our data supports the hypothesis that sympathetic hyperinnervation plays a role in the initiation of cardiopulmonary pathologies such as sudden cardiac death (SCD), arrhythmias, chronic respiratory failure, and chronic obstructive pulmonary disease.

Studies indicate that the cause of this hyperinnervation may be due to an elevation of nerve growth factor (Swissa et al. 2005; Verrier and Kwaku, 2004). Nerve growth factor is a neurotropin that supports the differentiation of Sympathetic neurons, enhances innervation, stimulates its own production within target organs, and determines the density of innervation by the Sympathetic nervous system (Levi-Montalcini, 1976; Korsching and Thoenen, 1983). Growth associated protein 43 (GAP43), a protein expressed in the growth cones of sprouting axons, is a marker for nerve sprouting. An increase of GAP43 positive nerves in dogs with atrial fibrillation suggests its involvement in sympathetic hyperinnervation (Chang et al. 2001).

Research has demonstrated an immediate elevation of transcardiac NGF concentration believed to be released by damaged myocardial cells post-MI (Heumann et al. 1984). Increased NGF following an MI enhances the sympathetic innervation to the viable myocardium, subsequently placing individuals at an increased risk for arrhythmogenesis. It is also believed systemic NGF supports a nerve sprouting signal within the LSG which creates a generalized increase in cardiac nerve density throughout noninfarcted myocardium and subsequently establishes sympathetic hyperinnervation (Zhou et al. 2004).

Our observation of an increased number of nerve cell bodies within the left stellate ganglia of cadavers with cardiopulmonary disease, in comparison to other pathologies, offers a possible link to sympathetic hyperinnervation noted in previously cited studies. Sympathetic regeneration through nerve sprouting is thought to cause the noted sympathetic hyperinnervation following a pathological disturbance.

Research suggests an increase in post-MI systemic NGF results in a retrograde transport of NGF to LSG which triggers nerve sprouting and sympathetic hyperinnervation—creating an environment for arrhythmia. The increase in NGF and GAP43 protein within the LSG without a concomitant increase in mRNA levels suggest a retrograde axonal transport of NGF from the infarcted site to the LSG (Korsching and Thoenen, 1988; Li et al. 1993). A possible mechanism is the release of NGF following a MI results in retrograde axonal transport of NGF to the left stellate ganglion resulting in nerve sprouting (Fig. 4).

**Figure 3.**
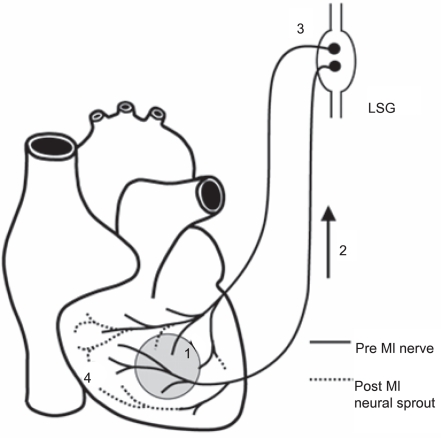
**Stellate ganglion involvement pathway**. Myocardial injury (shaded area) results in release of nerve growth factor (NGF), presumably from damaged cells, followed by upregulated NGF and growth-associated protein 43 (GAP43) expression (1). These signal proteins are then transported retrograde (2) to the nerve cell bodies of the stellate ganglia (3) where they stimulate nerve sprouting (4), predominantly in non-infarcted regions, leading to hyperinnervation (Image from Verrier and Kwaku, 2004).

**Figure 4.**
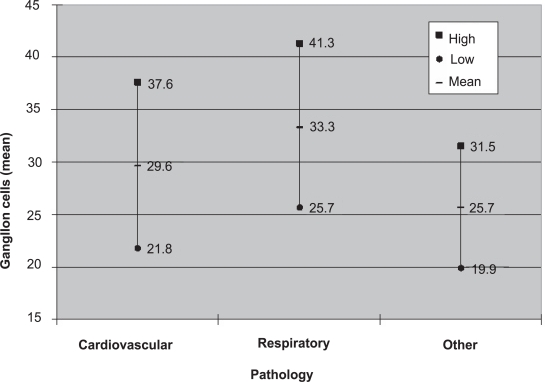
**Observed nerve cell bodies**. Mean and standard deviation of nerve cell bodies observed within the left stelliate ganglia.

**Figure 5.**
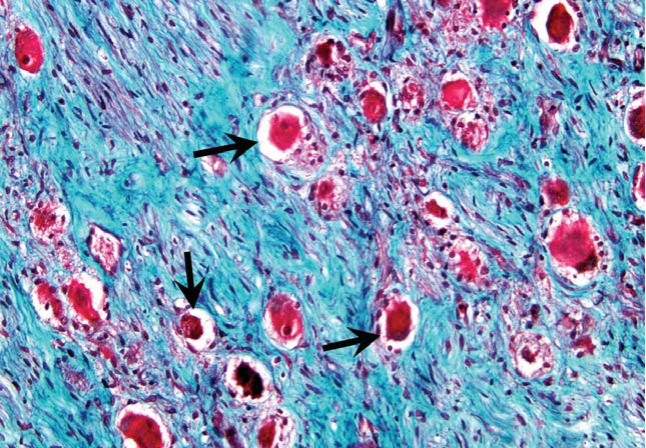
**Stellate ganglion section: “Other disease”**. Histological section of the left stellate ganglion stained with Masson’s trichrome stain. Sample was removed from a cadaver with a history of leukemia. Note a less prominent amount of nerve cell bodies (arrows).

Our observation of an increased number of nerve cell bodies within the left stellate ganglia of cadavers with cardiopulmonary disease offers a possible link to sympathetic hyperinnervation noted in previous studies. Histological section of a left stellate ganglion from an individual with coronary artery disease and AF demonstrates an increased number of nerve cell bodies (Fig. 3) as compared to a specimen from an individual without cardiopulmonary disease (Fig. 5). The increased number of nerve cell bodies observed in cadavers with cardiopulmonary pathology suggests NGF may initiate the generation of new ganglion cells which in-turn would cause an increased sympathetic tone to the distal organs. Though a P value of 0.199 was not statistically significant compared to other pathology, an increase in sample size in future studies may yield more statistically significant results. However, the observed increase in number of nerve cell bodies within the LSG presents a clinically significant result.

Stimulation of the sympathetic signal has also been implicated in respiratory diseases. Sympathetic hyperinnervation has been shown to cause an increase in pulmonary vascular resistance which is believed to a pathogenic factor in the development of pulmonary hypertension and cor pulmonale (Heindl et al. 2001). Increased sympathetic nerve activity has also been shown to play a vital role in advanced cases of pulmonary artery hypertension and decreases in arterial blood flow (Velez-Roa et al. 2004). Autonomic nervous system dysfunction has also been observed in patients with chronic obstructive pulmonary disease (COPD) as well (Volterrani et al. 1994; Scalvini et al. 1999; Bartels et al. 2000). One study suggests an increased constant sympathetic signal to the respiratory system is associated with increased airway resistance seen in COPD (Volterrani et al. 1994).

Similar to ischemic myocardial cells, human pulmonary epithelial cells and airway smooth muscle cells are a source of NGF during pulmonary pathological disease processes (Freund et al. 2002). Our finding of an increased number of nerve cell bodies in cadavers with respiratory pathology suggests a mechanism similar to cadavers with cardiovascular disease.

**Figure 6.**
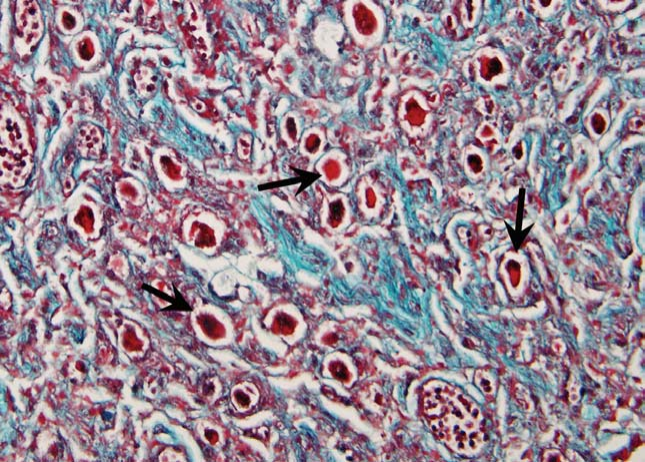
**Stellate ganglion section: Respiratory disease.** Histological section of the left stellate ganglion stained with Masson’s trichrome stain. Sample was removed from a cadaver with a history of lung cancer and COPD. Note increased number of nerve cell bodies (arrows).

Histological section of a left stellate ganglion from an individual with lung cancer and COPD demonstrates an increased number of nerve cell bodies (Fig. 6) as compared to a specimen from an individual without cardiopulmonary disease (Fig. 5).

An increased release of NGF may initiate an increase in nerve cell bodies of the LSG via a retrograde axonal transport causing sympathetic hyperinnervation to the respiratory system. A pathological change of the pulmonary system may cause a release of NGF, which may then return to the LSG via a retrograde axonal transport. The NGF within the LSG may cause sympathetic hyperinnervation to the pulmonary system which may subsequently cause an increase in morbidity and mortality of these individuals.

Our results are clinically significant considering the use of stellate ganglion blockade may provide protective benefits against post-MI arrhythmogenesis, SCD, and assist in such disease states as COPD and pulmonary hypertension. Considering infusion of NGF directly into the left stellate ganglion induced nerve sprouting and a higher incidence of ventricular tachycardia in dogs (Swissa et al. 2004), it would seem feasible that a left stellate block could reduce the sympathethic hyperinnervation noted in cardiovascular pathologies and prevent further morbidity and mortality. Previous literature has already demonstrated the ability of subacute left stellectomy to reduce the incidence of ventricular fibrillation following the onset of acute posterolateral ischemia (Nelson et al. 1989) and anterior myocardial infarction (Schwartz and Stone, 1980) using dog models.

Patients with pulmonary pathologies may also likely benefit from stellate ganglion block as well. Stellate ganglion block has been shown to be beneficial in decreasing pulmonary vein pressure and also causing a decrease in the amount of edema associated with lung tissue damage (Dauber and Weil, 1983). Based on this evidence, it would seem likely a stellate ganglion blockade would also assist in disease states such as COPD and pulmonary hypertension.

Though Horner’s Syndrome is a concern when performing any sympathetic block, thorascopy provides a magnified view of the sympathetic chain which can allow for precise surgical resection of the stellate ganglion and also reduce costs and length of hospital stays (Pather et al. 2006).

In our investigation, no statistically significant fibrotic changes were observed. We hypothesized obliteration of the nerve cell bodies by fibrotic changes may play a role in altering the sympathetic signal to distal tissues. However, this theory was not supported since no obliteration of nerve cell bodies by fibrotic changes was observed (Fig. 3).

Some study limitations do exist. First, the small sample size for each category (cardiovascular, n= 12: respiratory, n = 10, other n = 10) may influence the statistical signifance. A follow-up study with a larger sample size is warranted by our results in order to increase our statistical power. Secondly, the duration of time between onset of the pathology and death for each cadaver was unknown. Thus, it is unclear whether the number of nerve cell bodies noted on histological examination were caused by an acute or chronic conditions. Future studies with retrospective chart reviews of the cadavers or a prospective study following patients from disease onset to death and dissection would remedy this limitation.

## Figures and Tables

**Table 1.** Cadaver characteristics and pathology distribution.

	**Pulmonary**	**Cardiac**	**All-other**	**Total**
***N***	10	12	10	32
**Median Age (range)**	81 (73–92)	85 (56–98)	65 (28–93)	79
**Pathology Distribution**	Respiratory insufficiency	Cardiac Arrythmia; Cardiac Arrest	Upper GI hemorrhage, Gastric Ulcer	
	Emphysema	CHF^†^	Failure to Thrive	
	Metastatic Lung Cancer	Cardiac Arrest	Acute Myelogenous Leukemia	
	Pneumonia	CHF; CAD^‡^;	Metastatic Ovarian Cancer	
	Lung cancer; COPD*	Critical Aortic Stenosis; COPD; CAD	End Stage Renal Failure	
	Lung Cancer	CHF; Cardiac Arrest	Pancreatic Cancer	
	Respiratory Failure; Pleural Effusion	CAD; Atrial Fibrillation;	Malignant Brain Tumor	
	Non Small Cell Lung Cancer	Myocardial Infarction; Cardiogenic and Septic Shock;	Metastatic Prostate Cancer	
	Pneumonia; COPD	Cardiorespiratory Arrest; CAD	Hepatic Failure; Colon Cancer	
	Acute Respiratory Infarction	CAD; Myocardial Infarction CAD; Cardiorespiratory CAD	Head and Neck Cancer	

* Chronic obstructive pulmonary disease;

† Congestive heart failure;

‡ Coronary artery disease.
